# Microarray analysis reveals global modulation of endogenous retroelement transcription by microbes

**DOI:** 10.1186/1742-4690-11-59

**Published:** 2014-07-25

**Authors:** George R Young, Bettina Mavrommatis, George Kassiotis

**Affiliations:** Division of Immunoregulation, MRC National Institute for Medical Research, The Ridgeway, London, NW7 1AA UK; Department of Medicine, Faculty of Medicine, Imperial College London, London, W2 1PG UK

**Keywords:** Endogenous retrovirus, Retroelement transcription, Global transcriptional profiles, Microbial stimulation, Immune cells

## Abstract

**Background:**

A substantial proportion of both the mouse and human genomes comprise of endogenous retroelements (REs), which include endogenous retroviruses. Over evolutionary time, REs accumulate inactivating mutations or deletions and thus lose the ability to replicate. Additionally, REs can be transcriptionally repressed by dedicated mechanisms of the host. Nevertheless, many of them still possess and express intact open reading frames, and their transcriptional activity has been associated with many physiological and pathological processes of the host. However, this association remains tenuous due to incomplete understanding of the mechanism by which RE transcription is regulated. Here, we use a bioinformatics tool to examine RE transcriptional activity, measured by microarrays, in murine and human immune cells responding to microbial stimulation.

**Results:**

Immune cell activation by microbial signals *in vitro* caused extensive changes in the transcription not only of the host genes involved in the immune response, but also of numerous REs. Modulated REs were frequently found near or embedded within similarly-modulated host genes. Focusing on probes reporting single-integration, intergenic REs, revealed extensive transcriptional responsiveness of these elements to microbial signals. Microbial stimulation modulated RE expression in a cell-intrinsic manner. In line with these results, the transcriptional activity of numerous REs followed characteristics in different tissues according to exposure to environmental microbes and was further heavily altered during viral infection or imbalances with intestinal microbiota, both in mice and humans.

**Conclusions:**

Together, these results highlight the utility of improved methodologies in assessing RE transcription profiles in both archived and new microarray data sets. More importantly, application of this methodology suggests that immune activation, as a result of infection with pathogens or dysbiosis with commensal microbes, causes global modulation of RE transcription. RE responsiveness to external stimuli should, therefore, be considered in any association between RE transcription and disease.

**Electronic supplementary material:**

The online version of this article (doi:10.1186/1742-4690-11-59) contains supplementary material, which is available to authorized users.

## Background

While the existence of repetitive genetic elements has been recognized since the 1950s, the scale of their contribution to overall genome size was only fully realized through the sequencing of the human and mouse genomes [1, 2]. In total, repetitive elements comprise around 40% of both genomes, representing millions of years of accumulation. Over 90% of these sequences are retroelements (REs), replicating through a mechanism of reverse transcription. This group comprises long and short interspersed nuclear elements (LINEs and SINEs), and long-terminal repeat (LTR)-retroelements. The latter include endogenous retroviruses (ERVs) and mammalian apparent LTR-retrotransposons (MaLRs) that together comprise around 9% of both genomes [1, 2].

Originally identified as leukemogenic agents in mice, both exogenous and endogenous retroviruses have been extensively studied for potential contributions to cancer and disease in many species [3]. Many ERVs were integrated and fixed in the germ-line prior to many speciation events. During this time, they have suffered significant mutation, recombination, and deletion, and no infectious ERVs are currently recognized in the human genome [4]. The potential influence of ERVs polymorphic in the human population [5] is unknown, however, and ERVs and other REs are increasingly implicated in distinct physiological and pathological processes of the host [4, 6].

Dependent on their relative distance and orientation, REs have been suggested to act as transcriptional promoters and enhancers, canonical and alternative transcription initiation and termination points, splice donor and acceptor sites [7] and polyadenylation signals [8]. Further, there is increasing evidence that REs may be crucial components of the long intergenic non-coding RNA (lincRNA) regulatory system [9]. Over 80% of lincRNAs have been found to contain REs, which were enriched around the transcription start site of the transcript, suggesting a role in expression regulation [9].

Through co-option by the host, REs, and ERVs in particular, can have more direct effects. The fusogenic and immunomodulatory roles of certain ERV envelope sequences have been acquired as ‘syncytins’ separately in a variety of placental mammals [10]. Knock-out and knock-down studies have shown the crucial significance of these genes [11, 12]. More counterintuitively, endogenous retroviral sequences have also been co-opted to play roles in retroviral defense, as genes such as *Fv1* and *Fv4* [13–16].

Despite the lack of infectious ERVs in the human genome, ERV-encoded envelope glycoprotein antigens have been suggested as putative autoantigens in human autoimmune conditions and viral-like particles have been observed in a variety of human diseases [17, 18]. Complicating the establishment of causality, however, viral-like particles have also been noted in breast milk and tissues from healthy individuals, and can be induced from transformed cells from healthy donors [6]. Thus, while the potential impact of REs in infection and disease is a large area of current study, research is complicated by the scarcity of data describing their natural spatial and temporal patterns of transcription, and responsiveness to ubiquitous stimuli, including elements of diet [19]. An improved understanding of these areas is increasingly important given the recent identification of REs as potential vaccination targets in both cancer and human immunodeficiency virus-1 (HIV-1) infection [20].

Using mice with distinct immunodeficiencies, we have previously reported the spontaneous emergence and establishment of replication-competent murine leukemia viruses (MLVs) through recombination between replication-defective ERVs [21]. The appearance of infectious MLVs in immunodeficient mice was influenced by their exposure to environmental factors, most notably commensal microbes. It is possible that microbial stimulation induces the necessary expression of precursor ERVs, the first step in the recombination process, or the subsequent steps allowing the spread of these recombinant MLVs within and between animals. Although certain endogenous MLVs are known to be responsive to stimulation by microbial products, such as Toll-like receptor (TLR) agonists, ERV transcription is thought to be suppressed primarily by epigenetic silencing [22]. Whether the induction of ERVs by microbial stimulation is common or isolated remains unknown. To address this question, we have employed a microarray-based method that allows the determination of ERV expression more broadly. Using this method, we describe extensive patterns of ERV modulation by commensal or pathogenic microbes in both murine and human tissues.

## Results and discussion

### RE-reporting probes frequently follow the expression of their neighboring gene

Studies of RE transcription have to date relied primarily on PCR-based methods [23, 24], which has rendered techniques limited in scope to either expression analysis of individual loci or, conversely, to determination of generic, ‘family-wide’, expression patterns. Expressed sequence tag (EST) analysis [25] and customized spotted and, more recently, *in situ* synthesized microarrays [26, 27] have also been used to determine RE expression. However, such methodologies require specialized expertise or equipment, preventing their application in the majority of exploratory settings. Nevertheless, work with microarrays and related Northern-based approaches has so far revealed the potential for human ERV (HERV) induction by a variety of methods, including UV irradiation [28] and cytokine exposure [29].

While it has been known for some time that microarray platforms from various commercial manufacturers contain probe sequences corresponding to repetitive genetic elements, the major focus in the literature has been on the removal of such probes from analysis pipelines [30, 31]. Recently, reversal of this methodology, allowing the compilation of such probes, has been shown to facilitate determination of the genome-wide expression patterns of large numbers of diverse REs [32]. Previous work by Reichmann *et al.* [32] detailed a methodology designed to identify probes reporting RE expression. This methodology was updated in this study to utilize the latest version of the mouse and human genome sequences and extended to a larger set of microarray platforms. Marginally increased numbers of probes were identified, likely due to differences in the RepeatMasker [33] and RepBase [34] libraries used and the masking sensitivity. High levels of overall correspondence in identified probes were achieved with the previous study [32] (data not shown). Whilst cross-hybridization of microarray probes may potentially affect the assessment of expression of members of high-copy repeat families, large percentages (70-95%) of identified RE-reporting probes were mapped uniquely at a ≥ 95% identity level and thus likely reported the expression of single elements. Where probes were uniquely matched to the genome in this way, the distances to the nearest 3′ and 5′ genes, as well as their identities, were also recorded. 

**Figure 1:**
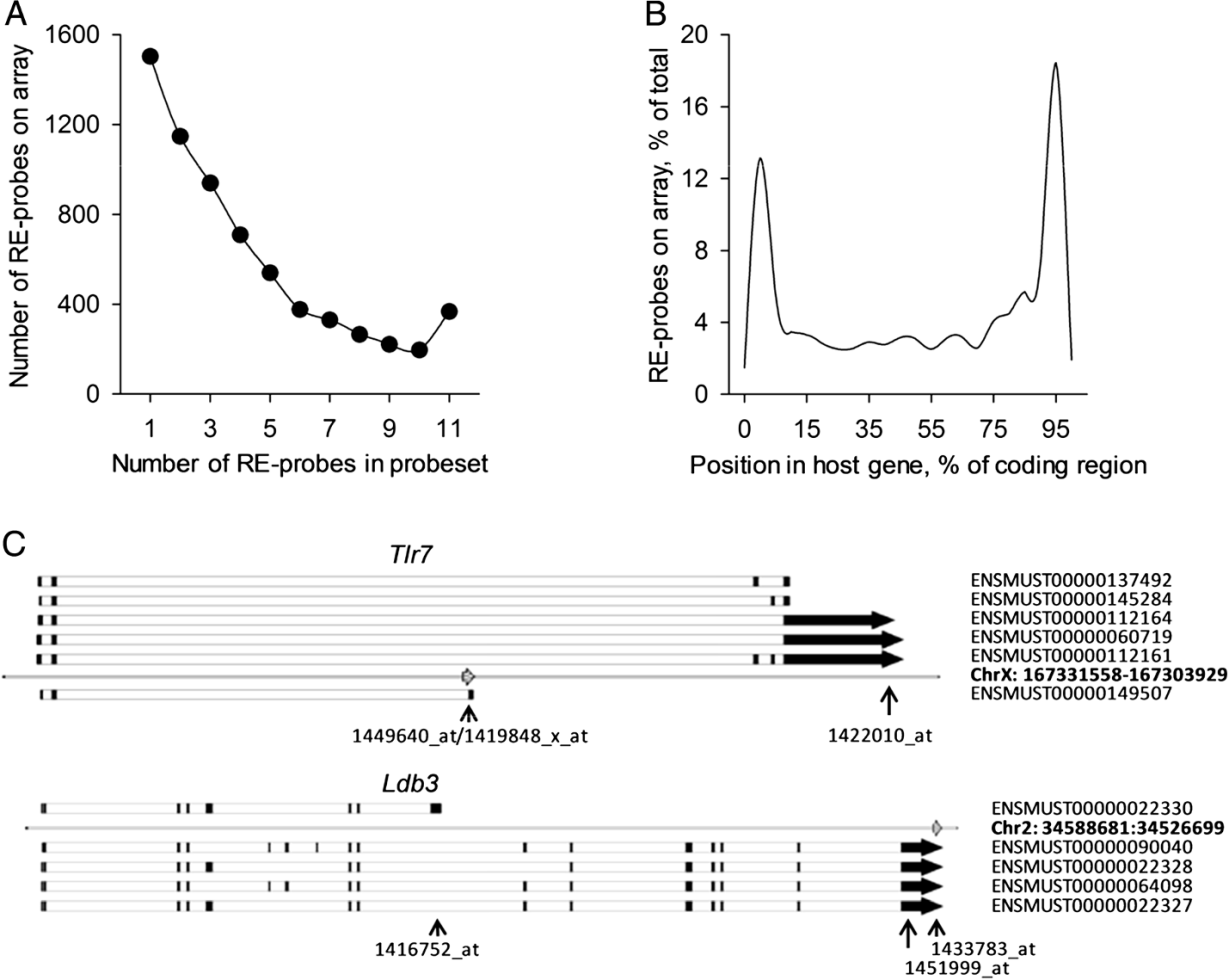
**Characterization of RE-reporting probes and their corresponding probesets for the Affymetrix Mouse Genome 430v2 microarray platform. (A)** Numbers of probesets (left axis) containing defined numbers of RE-reporting probes. The overall number of probesets containing RE-reporting probes was used to also express these values as a percentage of total (right axis). **(B)** Distribution of intragenic RE-reporting probes (including those within 1 kb of annotated genes) within the identified gene, expressed as a percentage of all RE-reporting probes within the platform. Locations are bins of percentage of gene length, to standardize for varying gene size. **(C)** mRNAs and their splicing patterns for two genes, represented by three probesets each, where a RE may be included in an alternate spice product (top) or within the canonical transcript (bottom). Track labeled with chromosome, location, and gene symbol shows the position and orientation of the reported RE (hashed arrow). Inclusion of the RE within an mRNA is denoted by its position either above (not included) or below (included) this track. Positions of probesets reporting the expression of the gene are shown below, with those in bold type containing RE-reporting probes. Data were obtained from the Ensembl Genome Browser.

Using the Affymetrix Mouse Genome 430v2 (MG430v2) platform, where a probeset was noted as containing RE-reporting probes, a median of 3 probes from the group were identified (Figure 1A). Only 12% of probesets identified consisted of a majority (>75%) of RE-reporting probes, however, and over 20% of probesets contained only a single RE-reporting probe (Figure 1A). Further, 68% of RE-reporting probes identified were within or immediately adjacent to annotated protein-coding genes (Figure 1B), raising the confounding factor that many REs reported may be co-regulated with neighboring genes, are included in canonical genic transcripts, or represented in mRNAs corresponding to alternative isoforms or splice variants (Figure 1C). This confounding factor broadly impacts analyses made with virtually any methodology used to date, excepting in instances where elements are successfully, specifically and uniquely targeted.

**Figure 2:**
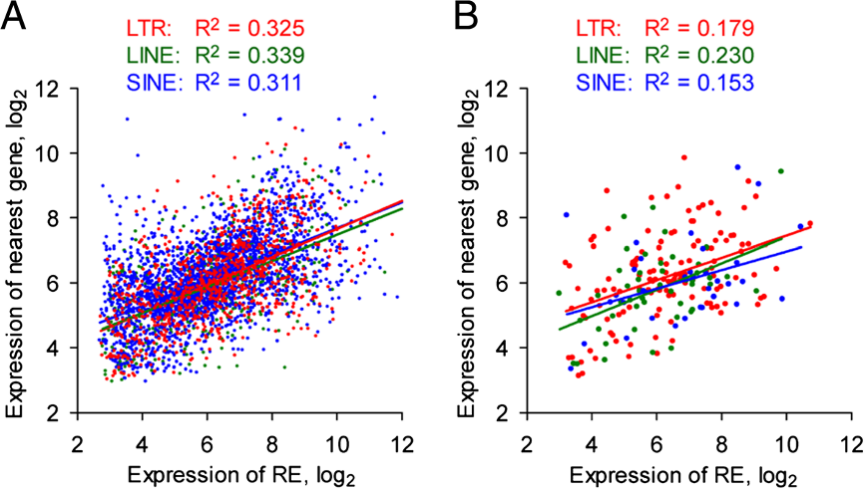
**Linkage of RE expression to activity of the nearest gene.** Regression of RE-reporting probe values for heart tissue samples against the one-step Tukey’s biweight *w*-estimator value calculated for all probes corresponding to all probesets for the nearest 5′ or 3′ gene, omitting points where the nearest gene was not present on the microarray platform. **(A)** All RE-reporting probes, as identified using previously published methodologies, and **(B)** RE-reporting probes passing enhanced filtering, that were significantly regulated between B6 tissues for three independent experiments using the Mouse Genome 430 v2 microarray platform (*p* < 0.001 by ANOVA comparing tissues and eliminating experiment). Data are obtained from E-GEOD-1986, −9954, and −10246.

To assess the potential impact of such co-regulation, three independent experiments using MG430v2, originally designed to determine tissue-specific expression patterns, were analyzed for significantly regulated RE-reporting probes. While obvious clustering of tissues was observed (data not shown), the most highly expressed RE-reporting probes were members of probesets reporting the expression of known tissue specific genes, including *Tnnt2* (*troponin T2, cardiac*) within heart tissue [35], *Ldb3* (*LIM domain binding 3*) within skeletal muscle [36], and *Ighv14-2* (*immunoglobulin heavy variable 14–2*) within the spleen [37]. Further supporting this observation, in a separate global analysis we found that when probesets contained a single RE-reporting probe, the behavior of the RE-reporting probe did not differ from that of the remainder of probes in the probeset across 9 tissues analyzed, in the vast majority of probesets (>86%) (p > 0.05, Holm-Bonferroni t test). To further investigate the extent of linkage between RE-reporting probe expression and that of a neighboring gene, correlation was assessed for heart tissue samples, which previously showed the greatest independence in RE-reporting probe expression. Varying significant (p < 0.0001) positive correlations were observed for LTR elements, LINEs and SINEs, suggesting expression patterns of neighboring genes explain ~30% of observed RE expression levels (Figure 2A).

While the differential regulation of RE-reporting probes in this manner may still have relevance, and indeed the transcriptional capacity of the RE may influence that of the gene, the independent regulation of REs within the genome cannot be easily assessed using this approach. To improve upon this, the published methodology was redesigned to increase stringency. Only RE-reporting probes from probesets that could be uniquely placed on the genome in a position intergenic to known protein-coding genes, and where >75% of probes were specific for a RE integration were retained. Numbers of probes passing this filtering are shown in Table 1.

**Table 1:** **Repetitive element representation within Affymetrix mouse microarrays**

Microarray platform	LTR	LINE	SINE	Total
**Murine genome u74a v2 affy_mg_u74a_v2**	243 (0.038)	79 (0.011)	37 (0.002)	359
**Mouse genome 430 2.0 affy_mouse430_v2**	2085 (0.330)	932 (0.141)	500 (0.033)	3517
**Mouse genome 430A 2.0 affy_mouse430a_v2**	3 86 (0.061)	94 (0.014)	45 (0.003)	525
**Mouse gene 1.0 ST affy_mogene_1_0_st**	1581 (0.250)	233 (0.035)	123 (0.008)	1937

Numbers of probes corresponding to LTR, LINE, and SINE elements across a subset of microarray platforms are shown. Shortened platform names correspond to identifiers used within the ‘oligo’ Bioconductor R package. Numbers in brackets indicate the estimated maximum percentage coverage of all individual LTR, LINE, or SINE elements by the microarray probes identified.

Tissue-specific RE expression patterns were again assessed using this filtering (Additional file 1: Figure S1). While considerably fewer RE-reporting probes were identified as differentially regulated, samples clustered according to tissue and, secondarily, by experiment (Additional file 1: Figure S1). Although all three groups exhibited robust tissue specificity, LTR elements represented the majority of REs that differed between tissues, followed by LINEs and then by SINEs (Additional file 1: Figure S1). This order reflected the representation of LTR, LINE and SINE elements on the microarray platforms, which favored LTR elements, whereas LINEs and, to a greater degree, SINEs were underrepresented (Table 1), likely due to their more repetitive nature in comparison with LTR elements.

The correlation between RE and neighboring gene expression was again assessed, with weaker positive correlations (p < 0.0219) being observed as the result of the enhanced filtering of RE-reporting probes (Figure 2B). In this analysis, LINEs displayed marginally higher degree of co-regulation with their nearest gene than either LTR elements or SINEs (Figure 2B). Thus, in addition to differences in their representation on the microarray platforms, LTR, LINE and SINE expression may involve divergent transcriptional mechanisms and linkage with neighboring genes. For these reasons, the remaining analyses focus solely on investigation of LTR elements, which were separated into the three classes recognized according to sequence similarity [38], with MaLRs included in class III.

### Assessment of RE expression in environmentally-exposed surfaces

**Figure 3:**
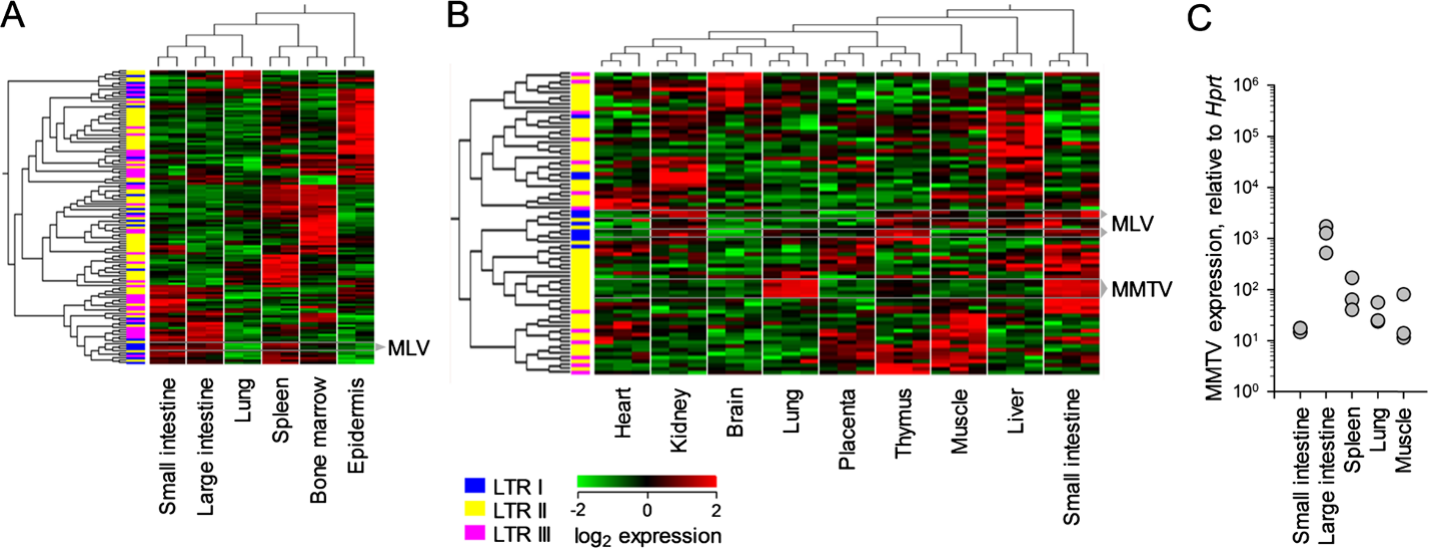
**Separate microarray platforms identify specific RE expression patterns in environmentally-exposed tissues.** Hierarchally-clustered heatmaps of RE-reporting probes significantly regulated between B6 tissues (*p* < 0.01 by ANOVA comparing tissues) for E-GEOD-10246 **(A)**, a Mouse Genome 430 v2 array, E-GEOD-97 and **(B)**, a Mouse Gene 1.0ST array. Where present, probes reporting expression of MLVs and MMTVs are highlighted. **(C)** qRT-PCR data detailing MMTV expression in tissues from B6 mice.

Previous work had outlined a potential role for husbandry conditions and the presence of commensal microbiota in influencing rates and probability of endogenous MLV recombination and subsequent emergence of infectious virus in variously immunodeficient mice on the commonly-used C57BL/6 (B6) genetic background [21]. To investigate this link further, a MG430v2 microarray dataset reporting expression patterns for environmental surfaces (lung, small and large intestine, and epidermis) was analyzed for RE expression (Figure 3A). Interestingly, all small and large intestine tissue samples showed elevated MLV expression. Expression in the intestinal tract was secondarily confirmed using an Affymetrix Mouse Gene 1.0 ST (MoGene1.0) dataset, which additionally showed in both the small intestine and lung high levels of mouse mammary tumor virus (MMTV) expression (Figure 3B), an ERV type not well represented in MG430v2. High levels of MMTV expression were confirmed in large intestine tissue samples by qRT-PCR (Figure 3C) using a methodology previously described [21], further supporting a potential link to microbial exposure in the control of ERV expression and validating the microarray data.

### ERV expression in the gut is dependent on both microbiota and genotype

Microbial products are recognized by pattern recognition receptors, such as TLRs, and previous work has shown the widespread and diverse impacts of various TLR agonists on ERV expression in both murine and human cells [21]. Subsequent to agonist recognition, TLR signaling converges through a limited number of downstream pathways, including, for many TLRs, a route including the Myd88 adapter molecule.

**Figure 4:**
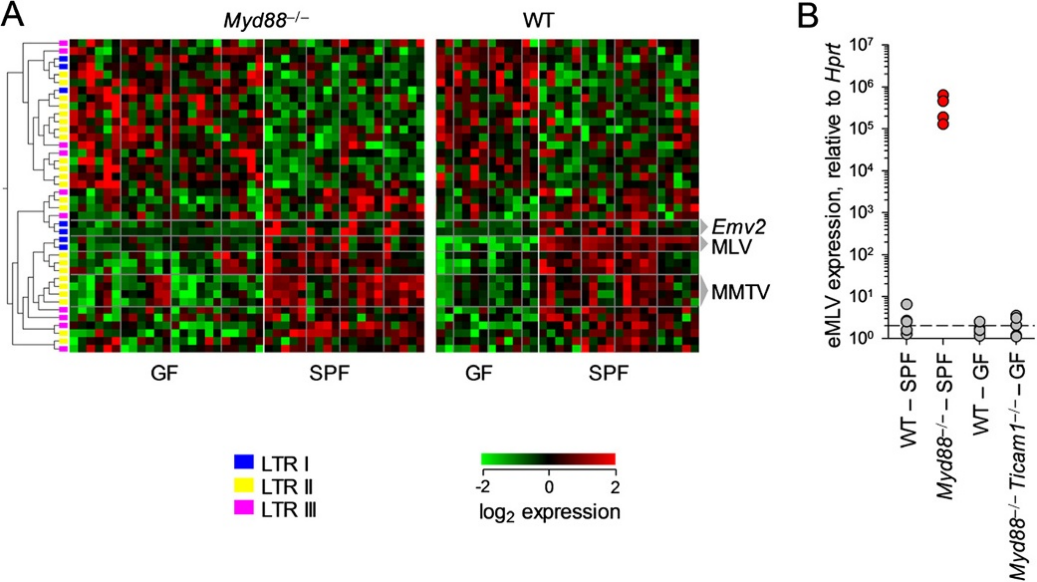
**RE expression in the gut is dependent on genotype and husbandry conditions. (A)** Heatmap of RE-reporting probes significantly regulated between GF and SPF housing conditions (*p* < 0.01 by ANOVA comparing husbandry conditions, eliminating genotype and tissue), using data from E-GEOD-17438, a Mouse Gene 1.0ST array. Each column is a single tissue from a single mouse. Intestinal tissues are separated with vertical lines in anatomical order: duodenum, jejunum, ileum and colon. Probes reporting MMTV, MLV, and *Emv2* expression are highlighted. **(B)** qRT-PCR analysis of eMLV expression between Myd88-deficient and -sufficient B6 mice housed in SPF or GF facilities. Values exceeding 10^3^ are considered high and are colored red.

To further investigate the dependence of ERV expression on the presence of a microbiota and on signaling from microbial products, the developed microarray methodology was applied to a MoGene1.0 array comparing a range of gut tissues from both wild-type and *Myd88*^−/−^ mice housed in both specific pathogen-free (SPF) and germ-free (GF) conditions (Figure 4A).

This analysis confirmed that, within wild-type mice, expression of certain RE families was dependent on the presence of the gut microbiota (Figure 4A). MLV expression, including that of the sole endogenous ecotropic MLV (eMLV) of B6 mice, *Emv2*, appeared entirely reliant on the presence of the microbiota. RLTR44-int (ERVK), MT2B (ERVL), and MMTV expression was also noticeably increased in SPF mice, albeit in tissue-specific manners (Figure 4A). A similar comparison within *Myd88*^−/−^ mice, while also showing largely decreased expression in GF housing conditions, also revealed the retention of some tissue-specific ERV regulation patterns. This included limited MLV expression within individual mice across multiple tissues (Figure 4A). A proportion of probes showed an opposing expression pattern, being elevated in tissues from GF mice, but represented various classes of REs, and no grouping was noted.

Comparison within SPF mice shows a marked effect of genotype, with significantly (p < 10^−7^) reduced MLV expression across all tissues sampled in the absence of Myd88 (Figure 4A). This finding suggested a role for Myd88 in the sensing of microbial stimuli that induced MLV expression specifically in SPF mice.

Together, these data supported a role for the microbiota and microbial signaling in elevating basal expression of both MLVs and MMTVs in the gut. We had previously linked the probability of recombinational rescue of *Emv2* to husbandry conditions, with no infectious virus being detectible in immunodeficient strains offered acidified water or maintained in entirely GF conditions. Interestingly, *Myd88*^-/-^ mice were an exception to this rule, maintaining some positivity when maintained with acidified water sources in various facilities [21]. GF *Myd88*^-/-^ mice were not available at the time to assess whether this viral rescue was, in fact, independent of the microbiota. To further investigate this question, therefore, wild-type and *Myd88*^−/−^*Ticam1*^−^^/-^ mice housed in GF conditions were compared with wild-type and *Myd88*^-/-^ controls maintained in SPF facilities (Figure 4B). No evidence of emergent virus was seen in GF *Myd88*^−/−^*Ticam1*^−^^/-^ mice.

Therefore, both the basal expression of MLVs and MMTVs in the gut, as well as the ultimate restoration of *Emv2* infectivity and the emergence of infectious recombinant MLVs rely on the gut microbiota in all strains tested.

### Microbial stimulation activates MLVs in a cell-autonomous manner

A recombinational rescue of *Emv2*, as previously noted in certain immunodeficient strains, would require transcription of not only the *Emv2* provirus, but concurrent and sufficient expression of a number of suitable recombination partners. These requirements, followed by the stochastic process of successful recombination, may act as a rate-limiting step in the production of infectious exogenous MLVs.

**Figure 5:**
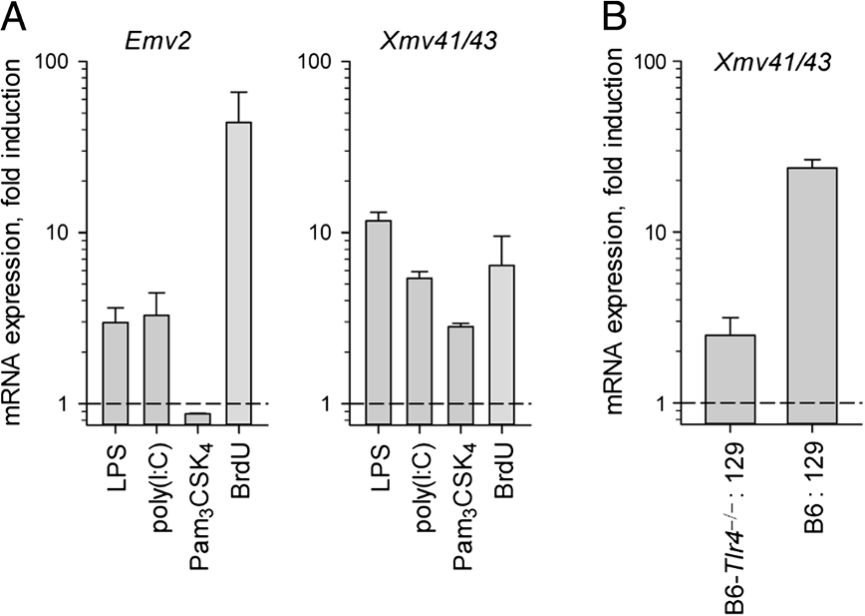
**TLR agonist-induced proviral expression is cell-intrinsic. (A)** qRT-PCR data showing fold induction of *Emv2* (left) and *Xmv41*/*43* (right) by TLR agonists (grey bars) or BrdU (blue bar) BMDCs. **(B)** qRT-PCR data showing fold induction of *Xmv41*/*43* in two cultures of mixed 129 and either *Tlr4*-sufficient or -deficient B6 BMDCs.

*Xmv43* (*Bxv1*), the expression of which is lipopolysaccharide (LPS)-inducible [39], was previously highlighted as a significant recombination partner in the rescue of *Emv2* [21]. The potential for stimulation with LPS or other TLR agonists to produce simultaneous expression of both proviruses was, therefore, examined in bone marrow dendritic cells (BMDCs) (Figure 5A). Expression levels were also compared to treatment with the halogenated thymidine analogue bromodeoxyuridine (BrdU), a treatment known to induce *Emv2* expression [40]. Treatment with both LPS, a TLR4 agonist, and polyinosinic-polycytidylic acid (poly(I:C)), a TLR3 agonist, significantly induced expression of both proviruses in culture, although no treatment with a TLR agonist matched the induction of *Emv2* seen upon BrdU treatment (Figure 5A). Treatment with Pam_3_CSK_4_, a TLR1/2 agonist, significantly induced *Xmv43* expression but caused a non-significant reduction in *Emv2* expression.

These data confirmed the possibility for TLR stimulation to cause the simultaneous expression of two viable recombination partners, but did not confirm that this occurred within the same cell. This requirement was investigated using co-culture of BMDCs produced from 129 mice, lacking *Xmv43*, and either wild-type or *Tlr4*^−/−^ B6 mice, retaining *Xmv43* but varying in their potential to respond to LPS stimulation (Figure 5B). Addition of LPS to co-cultures with *Tlr4*^−/−^ BMDCs gave only a small level of *Xmv43* induction, suggesting a minimal autocrine effect resulting from the stimulation of LPS-responsive 129 BMDCs. Significantly higher *Xmv43* induction was seen upon stimulation of co-cultures containing LPS-responsive wild-type B6 BMDCs (Figure 5B), however, suggesting that the majority of expression occurs in a cell-intrinsic manner.

### REs are significantly regulated on infection in both mice and humans

Recognition of pathogen-associated molecular patterns by pattern recognition receptors, such as TLRs, while perhaps a ubiquitous feature of the presence of commensals, is also more obviously associated with the detection of infection. Such signaling is crucial to the formation of appropriate defensive responses, and, alongside other pathways, can establish sustained differences in gene expression and protein production [41].

**Figure 6:**
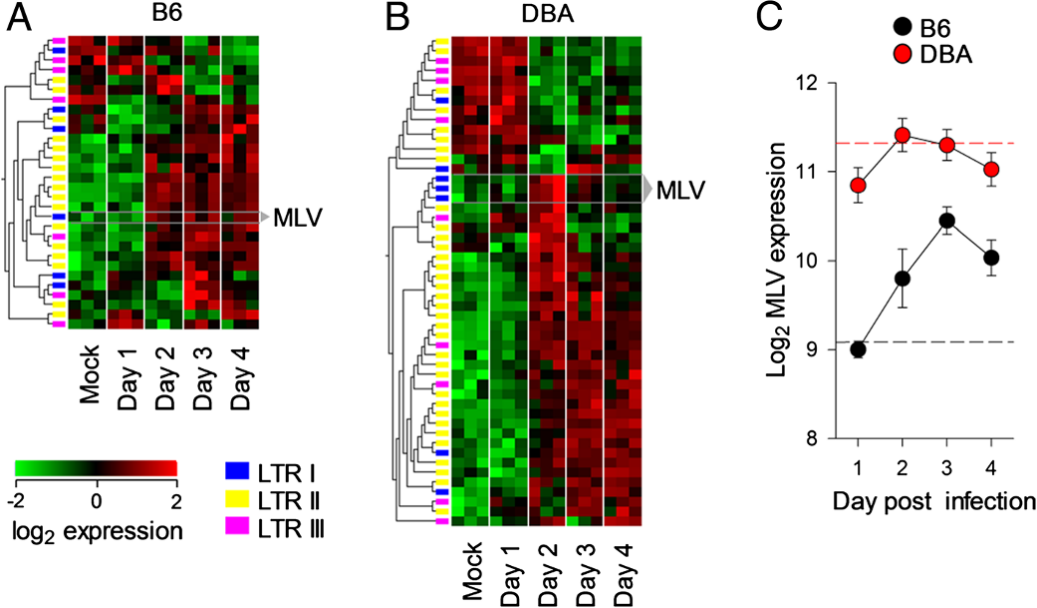
**RE expression in a murine influenza A model.** Heatmaps of significantly regulated RE-reporting probes during the first days of mouse influenza A infection (*p* < 0.01 by ANOVA comparing time point), for lung samples from B6 **(A)** and DBA2 **(B)** strains. Data are obtained from E-MTAB-835, a Mouse Genome 430 v2 array. Probes are hierarchally-clustered, whereas samples are ordered by time point. Probes reporting MLV expression are highlighted. **(C)** Median (±SEM) expression of MLV-reporting probes across all mice at each time point for B6 and DBA2 strains over the four days of the experiment. Hashed lines indicate the median of the mock-infected controls for B6 (black) and DBA2 (red).

To investigate the potential impact of viral infection on RE expression, microarray data examining influenza A infection in two strains of mice was analyzed. B6 and DBA2 mice, respectively resistant and susceptible to infection with influenza A, show differing immune responses [42], and, likewise, RE expression also varied (Figure 6A and B). Interestingly, B6 and DBA2 mice have different complements of all classes of endogenous MLV loci [43, 44], and display divergent expression patterns of MLV expression upon infection with influenza A. MLV induction within DBA2 mice was transient, appearing at day 2 post-infection before returning to baseline, whereas induction in B6 was sustained from day 2 post-infection for the duration of the experiment (Figure 6C). This difference likely not only reflects distinct programs of cellular gene expression, but also the particular responsiveness of individual proviral integrations.

While various factors may impact RE expression in mice, the complement, age, and degeneracy of REs and ERVs differs markedly between the mouse and human genomes. To allow comparisons to human datasets, the developed microarray methodology was extended to a variety of human microarray platforms (Table 2). HERV-K elements, subdivided into the HML-1 to −11 subgroups, contain the most recently endogenized proviruses within the human genome. Certain HERV-K(HML-2) proviruses remain polymorphic within the human population [5] and are suggested to be expressed in various situations, including upon HIV-1 infection [45–47]. The potential diagnostic or therapeutic relevance of HML-2 proviruses is a large area of current study, and, consequently, whilst the sequence similarity of these elements complicates the interpretation of expression measures (highly similar elements likely contribute, at least partially, but by an unquantified amount, to the expression observed for specific probes) the activity of HERVK-int, LTR5A, LTR5B, and LTR5_Hs elements was investigated where possible.

**Table 2:** **Repetitive element representation within Affymetrix human microarrays**

Microarray platform	LTR	LINE	SINE	Total
**Human genome u95a v2 affy_hg_u95a_v2**	191 (0.042)	95 (0.011)	0 (0)	286
**Human genome u133a v2 affy_hg_u133a_v2**	211 (0.046)	262 (0.030)	91 (0.006)	564
**Human genome u133 Plus v2 affy_hg_u133_plus_v2**	3022 (0.671)	2869 (0.337)	413 (0.027)	6304
**Human genome u219 affy_hg_u219**	503 (0.111)	398 (0.046)	42 (0.002)	943
**Human genome Focus affy_hg_focus**	18 (0.004)	71 (0.008)	9 (0.0006)	98
**Human gene 1.0 ST affy_hugene_1_0_st**	724 (0.160)	420 (0.049)	185 (0.012)	1329

Numbers of probes corresponding to LTR, LINE, and SINE elements across a subset of microarray platforms are shown. Shortened platform names correspond to identifiers used within the ‘oligo’ Bioconductor R package. Numbers in brackets indicate the estimated maximum percentage coverage of all individual LTR, LINE, or SINE elements by the microarray probes identified.

**Figure 7:**
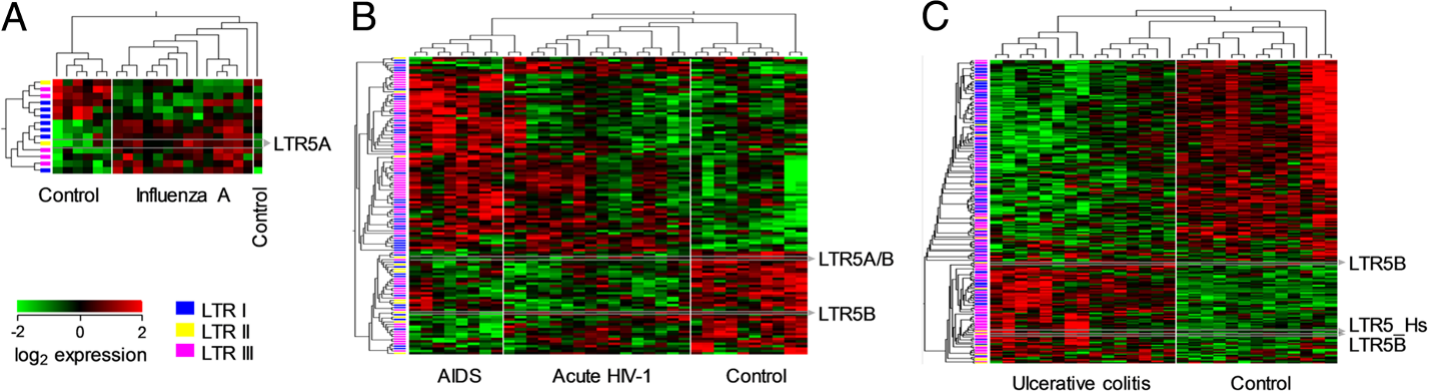
**RE expression in human disease.** Hierarchally-clustered heatmaps of RE-reporting probes significantly regulated between conditions (*p* < 0.01 by ANOVA comparing conditions and eliminating age and gender) for human influenza A **(A)**, HIV-1 infection **(B)**, and ulcerative colitis **(C)**. Respectively, data are from E-GEOD-6269 (a Human Genome U133A array sampling peripheral blood), and two Human Genome U133 Plus arrays, E-GEOD-16363 (sampling lymph node biopsies), and E-GEOD-38713 (sampling gut biopsies). Where present, probes corresponding to HML-2 elements are highlighted.

Previous work has identified the potential regulation of HERV-W family proviruses by influenza A [48]. To further translate the impact of influenza infection on the expression of murine REs to a human system, a comparative analysis of a human microarray dataset was made. This revealed a smaller effect of influenza infection (Figure 7A). Many fewer REs were significantly regulated, with similar numbers induced and repressed. The relatively small number of regulated elements found, whilst likely a factor of the size of the microarray platform used, may also be due to sampling peripheral blood, which might not reflect the full extent of disease activity in the target organ (lung). 

To investigate RE activity directly in an affected organ during viral infection, we applied the developed method on data from lymph node biopsies isolated from HIV-1-infected or uninfected individuals. Analysis of patients with acute HIV-1 infection or AIDS in comparison with healthy controls revealed a much larger number of significantly regulated elements (Figure 7B). Again, samples could be clustered effectively according to RE expression (Figure 7B).

Lastly, we examined if, similarly with their murine counterparts, expression of human REs and ERVs is influenced by exposure to microbial stimulation not only following infection, but also as a result of imbalanced homeostasis with gut microbes. Increasing volumes of research focus not only on the gut microbiome, but also on enteric fungal and viral constituents and the establishment and maintenance of gut immune homeostasis [49]. Fungal and viral patterns may also cause TLR stimulation, but are also recognized by a number of external pathways, which may act cooperatively or independently of TLRs. Dectin-1, for example, is suggested to allow the recognition of β-glucans, major constituents of the fungal cell wall [50]. To capture the complexity of such interactions, we compared human RE transcriptional profiles in gut biopsies from healthy individuals and ulcerative colitis patients. This analysis revealed extensive regulation, both induction and suppression, of a large number of REs in diseased tissue samples (Figure 7C). 

The potential regulation of HML-2 elements was investigated in all three cases, but low numbers of reporting probes prevent detailed analysis. A single HML-2-specific transcript reported by a LTR5A probe was upregulated in influenza A infection (Figure 7A). Transcripts reported by two probes (LTR5B and LTRBA/B) were modulated in acute HIV-1 infection and subsequent progression to AIDS (Figure 7B). Both of these were, however, reduced in abundance in infected individuals compared with uninfected controls (Figure 7B). In contrast, transcripts reported by three HML-2 specific probes (2 LTR5B and a LTR5_Hs) were significantly increased in ulcerative colitis samples in comparison with biopsies from healthy individuals (Figure 7C).

Thus, the analysis of tissues from individuals with viral infection or dysbiosis with intestinal microbiota demonstrated extensive modulation of RE activity, including members of the HML-2 family. However, due to the complex cellular composition of these tissues, combined with changes in this composition during infection or inflammation, these data did not allow determination of whether RE transcriptional changes were the result of genuine modulation in a specific cell-type or a side-effect of changing cellular composition of complex tissues. For example, the apparent decrease or increase of HML-2 activity in HIV-1 infection or ulcerative colitis samples, respectively, may simply represent the relative presence of lymphocytes or other hematopoietic cells in the tissue. Therefore, cell-intrinsic modulation of RE activity would require investigation of single cell types.

### Human RE transcriptional modulation by microbial stimulation is cell-intrinsic

**Figure 8:**
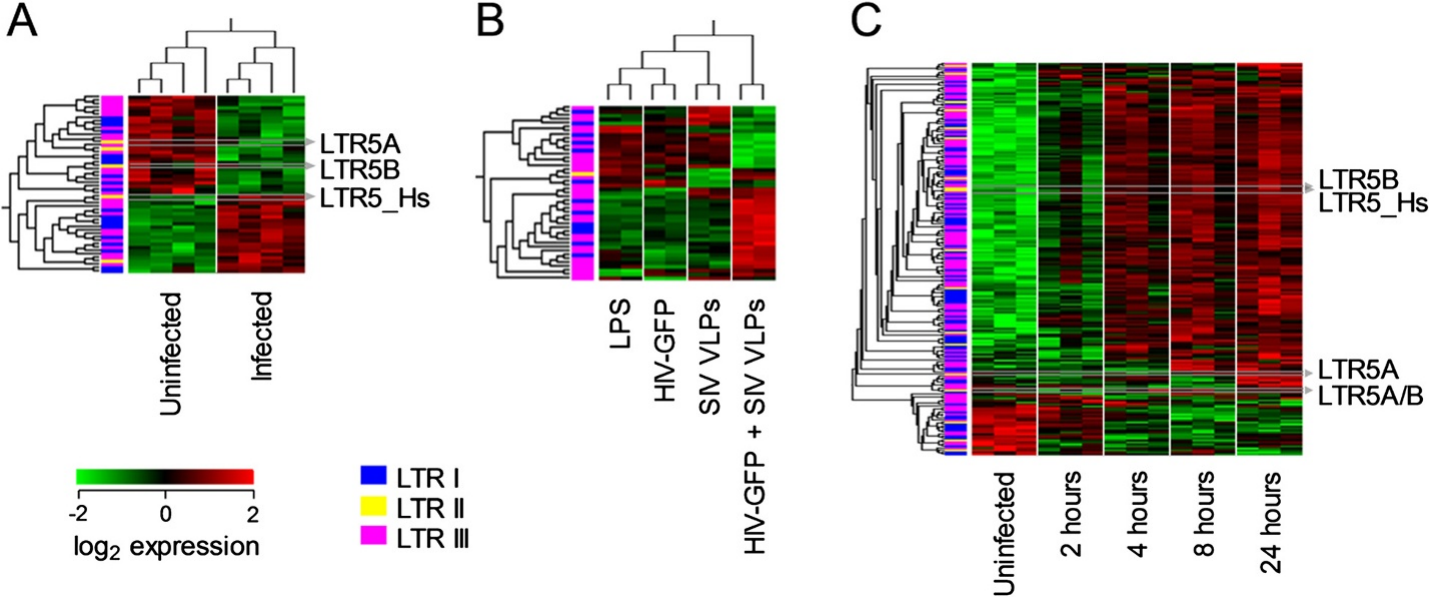
**RE expression in pure cell populations from in vivo and in vitro human infections.** Hierarchally-clustered heatmaps of RE-reporting probes significantly regulated (*p* < 0.01 by ANOVA comparing conditions) in **(A)** *ex vivo* HIV-1 infection (age and gender additionally eliminated from the ANOVA) and **(B)** *in vitro* HIV-1 infection. Data are from E-GEOD-42058 and −22589, Human Genome U133 Plus arrays, respectively. **(C)** Heatmap of significantly regulated RE-reporting probes in *Leishmania major* infected DCs (*p* < 0.01 by ANOVA comparing time points) ordered by time point with hierarchal clustering of probes. Data are from E-GEOD-42088, a Human Genome U133 Plus array. Where present, probes corresponding to HML-2 elements are highlighted.

To address this issue of cell composition in inflamed or healthy tissues, we analyzed the transcriptional activity of REs in specific human cell types either isolated *ex vivo* from human viral infection or exposed to microbial stimuli *in vitro*. The activity of several human REs was found altered in purified CD11c^+^ myeloid DCs isolated from peripheral blood mononuclear cells (PBMCs) of HIV-infected or uninfected individuals (Figure 8A). HML-2 transcripts reported by two of the three HML-2-specific probes that were found modulated in this comparison were downregulated in HIV-1 infection, whereas the third was upregulated (Figure 8A).

In a separate experiment, human DCs experimentally treated *in vitro* with HIV-1-based viruses and with Simian immunodeficiency virus (SIV) viral-like particles, a treatment that allows DC infection, exhibited an altered RE expression profile in comparison with all other treatment groups (Figure 8B), but no HML-2-specific probe was significantly regulated, potentially due to the omission of unstimulated control samples.

Lastly, human DCs *in vitro* infected with *Leishmania major* also considerably altered their RE expression profile, with numerous elements, including several HML-2 elements, significantly induced (Figure 8C). Induction of some REs appeared very rapid (2–4 hours), whereas other REs required prolonged stimulation (24 hours) (Figure 8C). Thus, direct microbial stimulation or infection of purified human immune cells causes extensive modulation of RE activity.

## Conclusions

Commercial microarray platforms contain thousands of RE-reporting probes, which can be used to assess RE transcriptional activity in a wealth of available data sets. However, these RE-reporting probes frequently correspond to REs that are near or within hosts genes and appear co-regulated with their nearest gene. Such co-regulation may be due to the capacity of REs to influence gene expression patterns within distinct cell types and to contribute to establishing the cell identity. It may also be partly due to the efforts of microarray manufacturers to focus on host gene transcription. Indeed, different microarray platforms detect certain RE families with variable coverage, and, therefore, the representation of REs in any one platform is incomplete. We further refined the microarray-based method to filter for RE-reporting probes identified as intergenic and as belonging to probesets where the majority of constituent probes report RE expression, to show global modulation of RE transcription at the level of individual cells or entire organs in both humans and mice exposed to microbial stimulation. As RE representation in this analysis is not complete, it is likely that the effect of microbial exposure on RE activity is even more extensive.

It is becoming clear that gene expression patterns are not fixed within cell types. Several cell types will respond to cues from other cells or the environment, and this is particularly true for immune cells responding to, for example an infection. Transcriptional reprogramming of immune cells also involves REs. In addition to immune cells tasked with sensing microbes, organs that are constantly exposed to the environment will express REs according to their microbial exposure. By being responsive to external stimuli, REs may not only participate in establishing the cell identity during development, but also help rewire gene expression networks to new patterns, ones that underlie the cellular response to these external stimuli.

## Methods

### Identification of probes reporting retroelement expression

The GRCm38.72 and GRCh37.72 releases of the mouse and human genomes were downloaded with accompanying gene annotation files and local BLAST + databases were constructed using BLAST 2.2.28+. RepeatMasker 4.0.3 (configured with TRF 4.04 [51] and RMBLASTn 2.2.28+ alongside the 20120418 RepBase library [52]) was used to mask both genomes using the ‘-s’ (sensitive) parameter. Microarray probe sequences and unique identification numbers were obtained either from annotation databases supplied for use with the ‘oligo’ [53] microarray analysis Bioconductor [54] package or from the manufacturer’s website.

A Python (http://www.python.org) script was produced to run and query BLASTn of the downloaded probes against the relevant genome using the ‘-task blastn-short’ parameter. The number of times an individual probe could be localized to the genome with ≥95% identity was recorded, along with the location of the highest scoring hit. A further Python script was used to parse these data to identify probes falling entirely within regions masked by RepeatMasker and to identify those in the correct orientation to report sense expression of the particular element. For technologies hybridizing antisense cRNA (e.g. Affymetrix 3′ microarrays), probes are sense to the retroelement, whereas for technologies hybridizing sense cDNA (e.g. Affymetrix Gene microarrays), probes are required to be antisense to the retroelement. The nearest genes chromosomally 5′ and 3′, as well as their locations, were recorded from the gene annotation files and, together, this information was compiled to form an annotation file for probes identified as reporting retroelement expression. Where probes were originally identified as reporting expression from multiple genomic loci, annotation information requiring a specific genomic context was omitted. This probe list was filtered using an additional script for probes derived from probesets where >75% of probes report retroelement expression, and where the probe was identified as >1 kb from the nearest protein coding gene. Annotation files are supplied as Additional files 2 and 3.

### Analysis of Affymetrix microarray data

Raw CEL files corresponding to accessions E-GEOD-97 [55], E-GEOD-1986, E-GEOD-6269 [56], E-GEOD-9954 [57], E-GEOD-10246 [58], E-GEOD-16363 [59], E-GEOD-17438 [60], E-GEOD-22589 [61], E-GEOD-24940 [62], E-GEOD-38713 [63], E-GEOD-42058 [64], E-GEOD-42088, and E-MTAB-835 [42] were downloaded from ArrayExpress (http://www.ebi.ac.uk/arrayexpress). Pseudo-images of the array chips were visually inspected for spatial artifacts and arrays that passed this inspection were analyzed at the probe level with a custom R script utilizing routines provided within ‘oligo’. Perfect-match (PM) probe expression data for the entire dataset were RMA background corrected and quantile normalized before log2 transformation and export. Downstream analysis, probe annotation, batch-effect correction (where appropriate), and heatmap production was thereafter performed with Qlucore Omics Explorer (Qlucore, Lund, Sweden). To reduce the size of heatmaps and to decrease artificial clustering resulting from multiple probes from the same probeset, probes identified as significant were collapsed into their respective probesets using facilities build into Qlucore Omics Explorer.

Other figure production and statistical analysis was performed with SigmaPlot v12 (Systat Software Inc, San Jose, CA, USA).

Calculation of the one-step Tukey’s biweight *w*-estimator for probeset expression followed the algorithms defined by Affymetrix [65]. For a number,*N*, of probe expression values, *x*, where  denotes the median of *x*, and *S* denotes the median absolute deviation of *x*, the *w*-estimator is calculated as , where  and , given the fixed values *c* = 5 and ϵ = 0.0001.

### Mice

Inbred B6 and 129 wild-type strains, as well as B6-backcrossed MyD88-deficient B6.129P2-*Myd88*^*tm1Aki*^ (*Myd88*^−/−^) and TLR4-deficient B6.129P2-*Tlr4*^*tm1Aki*^ (*Tlr4*^−/−^) mice have been described [66, 67]. Mice were bred in individually ventilated cages (IVCs) before being transferred to SPF facilities at the NIMR, and maintained on UV-irradiated, filtered neutral pH water. B6 and B6.129P2-*Myd88*^*tm1Aki*^*Ticam1*^*tm1Aki*^ (*Myd88*^−/−^*Ticam1*^−/−^) mice, additionally deficient for toll-like receptor adaptor molecule 1 (TICAM-1) [68], were also maintained in germ-free facilities at the Unit for Laboratory Animal Medicine, University of Michigan, MI, USA (UMICH) and kept on autoclaved distilled water. Animal experiments were approved by the ethical committee of the NIMR, and conducted according to local guidelines and UK Home Office regulations under the Animals Scientific Procedures Act 1986 (ASPA) and the authority of Project License PPL 70/7643.

### Cell culture

For the production of BMDCs, bone marrow was flushed from the femurs and tibiae of culled mice and incubated in IMDM supplemented with 5% FCS (Sigma-Aldrich, St Louis, MO, USA) and 10% GM-CSF for 7 days at 37°C and 5% CO_2_. Adherent DCs could typically be obtained after this time at a purity of 50-70%. TLR agonists were introduced for 48 hours at 1 μg/ml for LPS (from *Salmonella minnesota* R595, Axxora, CA, USA), 10 μg/ml for poly(I:C) (Sigma-Aldrich) and 0.25 μg/ml for Pam_3_CSK_4_ (Axxora). BrdU (Sigma-Aldrich) was introduced at 20 μg/ml.

### qRT-PCR and microarray analyses

Prior to cDNA preparation, all samples were stored in RNAlater (Qiagen, Hilden, Germany) at −20°C. Where tissues were processed, samples were disrupted using a TissueLyser LT (Qiagen). RNA was extracted from samples using RNeasy spin columns (Qiagen) and extracted nucleic acids were subjected to DNaseI (Qiagen) treatment in solution and a further column cleanup. RNA for qRT-PCR was reverse transcribed using the Applied Biosystems (Carlsbad, CA, USA) high capacity reverse transcription kit with an added RNase-inhibitor (Promega Biosciences, Madison, WI, USA) and cDNA was cleaned using QIAquick spin columns (Qiagen). All elutions were conducted with nuclease-free water (Qiagen).

Purified cDNA was used as template for the amplification of target gene transcripts with SYBR Green PCR master mix (Applied Biosystems) using the ABI Prism SDS 7000 and 7900HT machines (Applied Biosystems). Target gene expression was determined relative to *Hprt* using the ΔCT method using previously-described primer sets and methodology [21]. In plots showing expression, a hashed line indicating the theoretical detection limit is shown. Fold change values are calculated against an unstimulated control, represented by the hashed line, which is standardized to 1.

## Authors’ information

GRY and BM are post-doctoral Career Development Fellows in GK’s laboratory. GK is a program leader at MRC National Institute for Medical Research, UK, and Professor of Retrovirology at Imperial College London, UK.

## Electronic supplementary material

Additional file 1: Figure S1: Tissue-specific RE expression patterns. Hierarchally-clustered heatmap of RE-reporting probes significantly regulated between B6 tissues for three independent experiments using the Mouse Genome 430 v2 microarray platform (*p* < 0.001 by ANOVA comparing tissues and eliminating experiment). Data are obtained from E-GEOD-1986, −9954, and −10246, which are identified with numbers. (PDF 6 MB)

Additional file 2: **Archive of mouse annotation files. Probe annotation files (csv format), as defined in the Methods, for the following Affymetrix platforms: mg_u74a, mg_u74a_v2, mg_b74b, mg_u74b_v2, mg_b74c, mg_u74c_v2, moe_430a, moe_430b, ht_mg_430a, mogene_1_0_st, mogene_2_0, mouse430_v2, mouse430a_v2.** Shortened platform names correspond to identifiers used within the ‘oligo’ Bioconductor R package. Column identifiers are pid – probe id, probeset – Affymetrix probeset, plen – probe length, sid – target chromosome, sstart – start position of probe on sid, send – end position of probe on sid, nident – identity within the region sstart to send, numhits – number of hits recorded by BLASTn, repeat – RepBase-defined repeat, repclass – RepBase-defined repeat class, rstart – start position of repetitive element, rend – end position of repetitive element, 5id – symbol of nearest 5′ protein coding gene, 5start – start position of 5id, 5stop – end position of 5id, 3id – symbol of nearest 3′ protein coding gene, 3start – start position of 3id, 3stop – end position of 3id. (ZIP 846 KB)

Additional file 3: **Archive of human annotation files.** Probe annotation files (csv format), as defined in the Methods, for the following Affymetrix platforms: hg_u95a, hg_u95a_v2, hg_u95b, hg_u95c, hg_u95e, hu6800, hg_u133a, hg_u133a_v2, hg_u133b, hg_u133_plus_v2, hg_u219, hg_focus, hugene_1_0_st, and hugene_2_0_st. Shortened platform names correspond to identifiers used within the ‘oligo’ Bioconductor R package. Column identifiers are pid – probe id, probeset – Affymetrix probeset, plen – probe length, sid – target chromosome, sstart – start position of probe on sid, send – end position of probe on sid, nident – identity within the region sstart to send, numhits – number of hits recorded by BLASTn, repeat – RepBase-defined repeat, repclass – RepBase-defined repeat class, rstart – start position of repetitive element, rend – end position of repetitive element, 5id – symbol of nearest 5′ protein coding gene, 5start – start position of 5id, 5stop – end position of 5id, 3id – symbol of nearest 3′ protein coding gene, 3start – start position of 3id, 3stop – end position of 3id. (ZIP 3 MB)

## Abbreviations

B6: C57BL/6
BMDC: Bone marrow dendritic cell
BrdU: Bromodeoxyuridine
eMLV: Ecotropic MLV
ERV: Endogenous retrovirus
EST: Expressed sequence tag
GF: Germ-free
HERV: Human ERV
HIV-1: Human immunodeficiency virus 1
lincRNA: Long intergenic non-coding RNA
LINE: Long interspersed nuclear element
LPS: Lipopolysaccharide
LTR: Long-terminal repeat
MLV: Murine leukemia virus
MMTV: Mouse mammary tumor virus
PBMC: Peripheral blood mononuclear cell
poly(I: C): Polyinosinic-polycytidylic acid
RE: Retroelement
SINE: Short interspersed nuclear element
SIV: Simian immunodeficiency virus
SPF: Specific pathogen-free
TLR: Toll-like receptor
