# Supplementary material for: Microarray analysis reveals global modulation of endogenous retroelement transcription by microbes
Source: Retrovirology. 2014 Jul 25;11:59. doi: 10.1186/1742-4690-11-59 (PMC4222864; doi:10.1186/1742-4690-11-59)
Supplement: Supplementary file 1 — Additional file 1: Figure S1: Tissue-specific RE expression patterns. Hierarchally-clustered heatmap of RE-reporting probes significantly regulated between B6 tissues for three independent experiments using the Mouse Genome 430 v2 microarray platform (p < 0.001 by ANOVA comparing tissues and eliminating experiment). Data are obtained from E-GEOD-1986, −9954, and −10246, which are identified with numbers. (PDF 6 MB) [file 12977_2014_3913_MOESM1_ESM.pdf]

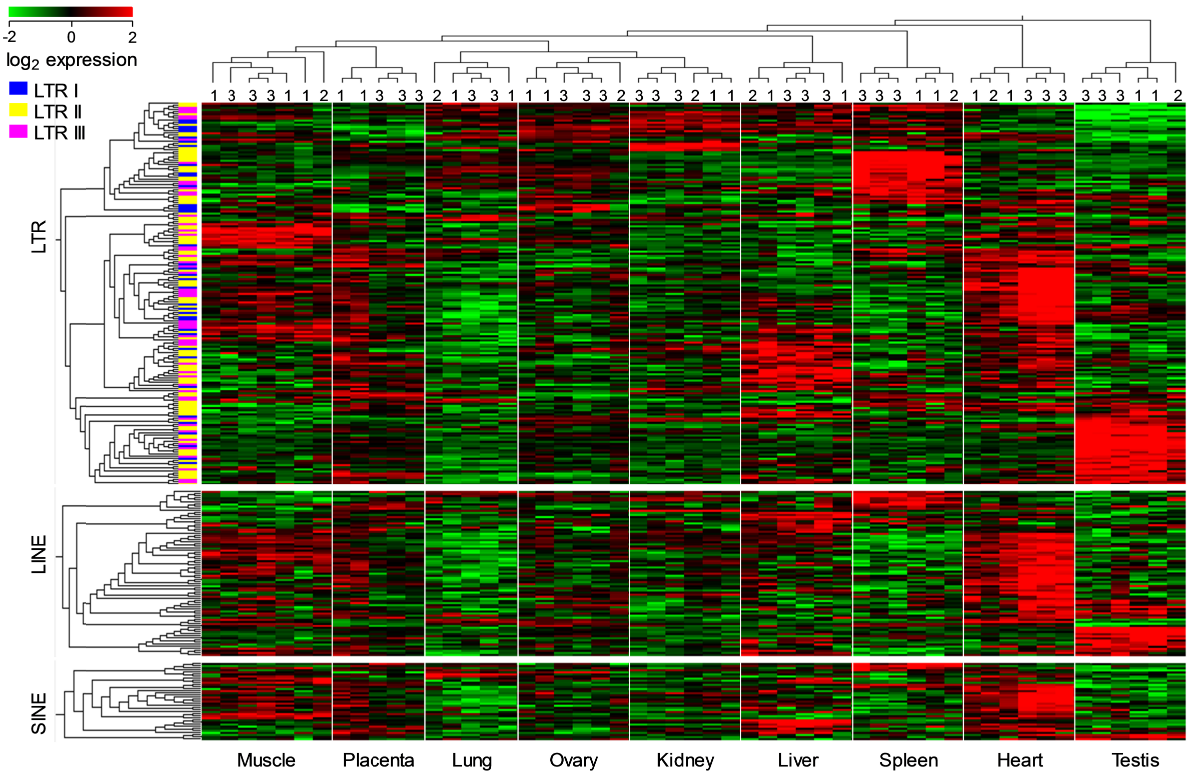

Figure S1 Tissue-specific RE expression patterns

Hierarchally-clustered heatmap of RE-specific probes significantly regulated between B6 tissues for three independent experiments using the Mouse Genome 430 v2 microarray platform (p<0.001 by ANOVA comparing tissues and eliminating experiment). Data are obtained from E-GEOD-1986, -9954, and -10246, which are identified with numbers 1, 2 and 3, respectively.
